# More Than a Motivational Poster

**DOI:** 10.31486/toj.21.5020

**Published:** 2021

**Authors:** Ronald G. Amedee

**Affiliations:** Head of Clinical School and Professor, The University of Queensland Faculty of Medicine, Ochsner Clinical School, New Orleans, LA; Editor-in-Chief, *Ochsner Journal*

*Keep Calm and Carry On*

The fall 2021 edition of the *Ochsner Journal* contains 6 original research articles, a quality improvement paper, a single review and contemporary update, and 8 case reports and clinical observations.

Considering works under the original research heading, you will find a topic near and dear to many readers in our area on “Impact of New Orleans Saints Football Games on Internal Medicine Admissions” by Nguyen and Chakraborti which appears just after the launch of the new NFL season. A major topic in health care being addressed at this time is the opioid crisis, and Eshraghi, Hanks, Rooney et al focus on a clinically specific issue: “Establishing a Dose-Response Relationship Between Opioid Use and Hypogonadism: A Retrospective Case-Control Study.” “Preoperative Administration of Hycet Elixir Reduces Hospital Length of Stay After Pediatric Outpatient Adeno/Tonsillectomy” from the Ochsner Department of Anesthesia provides intriguing evidence for a simple intervention that can improve patient care. Rounding out this section is the article by Mahato, Ganesh, Hanumanthu et al “Implementation of the 2017 American College of Cardiology/American Heart Association Guidelines on Hypertension in Clinical Practice.”

In the quality improvement section of this edition, Brody-Camp, Parsel, Freeman et al offer “Decreased Complications After Total Laryngectomy Using a Clinical Care Pathway,” and in the reviews and contemporary updates section, please find “Directed Donation: Special Considerations and Review for Contemporary Clinical Practices” by Wadge, Zhang, Seal et al.

The case reports and clinical observations section contains 2 papers on the successful use of stereotactic body radiotherapy for oligometastasis; one case details the treatment of metastatic prostate cancer, the other concerns contralateral kidney oligometastasis from renal cell carcinoma. Other papers in this section include a report from Robles-Torres, Arrambide-Herrera, Molina-Ayala et al on “Collision Tumor of the Kidney: Renal Cell Carcinoma Hidden in a Giant Angiomyolipoma in a Patient with Tuberous Sclerosis Complex,” “A Peptide Meets a Radionuclide to Combat a Rare Tumor” by Thomas, Boudreaux, Thiagarajan et al, and “Exercise Stress Test–Induced Atrioventricular Dissociation with Syncope” by Oo, Bhavsar, Aung et al.

Our quarterly Sports Medicine column by Fahy, Miller, Kobayashi et al is “Efficacy of Platelet-Rich Plasma on Symptom Reduction in Patellar Tendinopathy.”

The phrase *Keep Calm and Carry On* is frequently seen today on T-shirts and coffee mugs, but its origin was as a motivational poster created by the British government in 1939 to prepare its citizens for World War II. Nearly 2.5 million copies of the poster were made and distributed to the British people to boost morale as the country prepared for massive bombings of their major cities. As this introduction is being written in late July, the state of Louisiana is leading the country in a fourth COVID-19 surge largely due to the Delta variant and the low vaccination stats in comparison to many other states in the union. In essence, all members of our Ochsner health care team have once again gone to “war” against this continuing pandemic. Thank you all for your professionalism, courage, and resilience that throughout this pandemic have been on full display. This fourth surge is largely one of the unvaccinated, and we still have much work to do in our collective efforts to bring herd immunity to the citizens of Louisiana.

